# Effects of Thermal Aging on Optical, Surface, Mechanical, and Biological Properties of CAD/CAM Polymer-Based Dental Materials

**DOI:** 10.3390/polym18060760

**Published:** 2026-03-20

**Authors:** Gül Ateş, Mustafa Gungormus

**Affiliations:** 1 Department of Prosthodontics, Faculty of Dentistry, Ankara Yıldırım Beyazıt University, Ankara 06010, Türkiye; 2 Department of Basic Sciences, Faculty of Dentistry, Ankara Yıldırım Beyazıt University, Ankara 06010, Türkiye

**Keywords:** CAD/CAM polymers, PEEK, fiber-reinforced composites, graphene-reinforced polymers, thermal aging, dental materials

## Abstract

CAD/CAM polymer-based dental materials are increasingly used as metal-free alternatives for fixed and implant-supported restorations. High-performance polymers such as polyetheretherketone (PEEK), fiber-reinforced composites, and graphene-reinforced polymers have been introduced to improve material stability; however, evidence regarding the effects of thermal aging on their physicochemical and biological properties remains limited. In this study, PEEK, a fiber-reinforced composite (FRC), and a graphene-reinforced PMMA-based polymer (G-CAM) were evaluated. Twenty-seven disc-shaped specimens (10 × 2 mm; *n* = 9 per material) were fabricated and subjected to 10,000 thermal cycles between 5 and 55 °C. Color change (ΔE_00_), surface roughness (Ra), and Vickers microhardness (VHN) were measured before and after aging. Chemical stability was assessed using FTIR and Raman spectroscopy, surface morphology by SEM analysis, and biological safety by cytotoxicity testing. Material-dependent differences were observed in color stability, surface roughness, and microhardness after thermal aging (*p* < 0.05). Microhardness decreased in the fiber-reinforced and graphene-reinforced materials, whereas PEEK showed no significant change. Spectroscopic analyses indicated preserved chemical structure, and all materials demonstrated acceptable cytocompatibility. Thermal aging influenced material behavior while chemical stability and biological safety were maintained, highlighting the importance of considering aging behavior during material selection for prosthetic restorations.

## 1. Introduction

The rapid development of computer-aided design and computer-aided manufacturing (CAD/CAM) technologies has changed restorative dentistry by enabling the digital fabrication of prosthetic restorations under standardized industrial conditions. Compared with conventional resins, CAD/CAM polymer blocks provide improved structural homogeneity, lower internal porosity, and better reproducibility. These characteristics support more predictable mechanical performance and clinical reliability [1,2]. However, restorative materials are continuously exposed to intraoral conditions, including cyclic occlusal loading, moisture, and temperature changes. Over time, these factors can affect mechanical integrity and alter surface characteristics [3,4]. Conventional polymethyl methacrylate (PMMA)-based resins are widely used because of their aesthetic properties, low cost, and ease of processing. However, they exhibit limitations, including low fracture resistance, wear behavior, and reduced long-term durability [5,6]. These mechanical limitations have led to the development of high-performance polymer-based materials [7].

In recent years, polymer-based CAD/CAM materials have been used as alternatives to metal and ceramic frameworks for implant-supported and fixed prosthetic restorations. Among these materials, polyether ether ketone (PEEK) is gaining attention in prosthetic dentistry. PEEK is a semi-crystalline thermoplastic polymer belonging to the polyaryletherketone family and is a chemically stable, corrosion-resistant, and biocompatible material. Thanks to its elastic modulus, which is closer to that of cortical bone than traditional metal alloys, it contributes to improved stress distribution in implant-supported restorations [8]. However, its mechanical strength is lower than that of zirconia and metals.

Despite its increasing clinical adoption, scientific evidence regarding the long-term physico-mechanical and optical behavior of PEEK-based CAD/CAM materials under simulated intraoral aging conditions remains limited. Aging-related conditions, such as water sorption–induced plasticization and thermal cycling, are considered critical mechanisms that influence the surface integrity, mechanical performance, and optical stability of PEEK materials during clinical use [9,10]. Therefore, further investigations evaluating contemporary polymer-based CAD/CAM materials under standardized aging protocols are needed to clarify their long-term clinical performance.

Fiber-reinforced composites (FRC) are another high-performance material in which the polymer matrix is reinforced with multi-directional glass or polymeric fibers. In these materials, the reinforcement phase increases load-carrying capacity and helps limit crack propagation, while the surrounding matrix facilitates stress transfer and supports CAD/CAM machinability [11,12]. These materials have been proposed as durable alternatives to rigid ceramic frameworks, particularly in situations where shock absorption and high stress modulus are desired.

More recently, nanographene-reinforced PMMA CAD/CAM polymers have been introduced by incorporating graphene-derived nanofillers into thermoplastic acrylic matrices. Graphene incorporation has been associated with improved mechanical performance, increased crack resistance, and favorable optical and surface properties, while maintaining the flexibility and workability of polymer materials [6,10]. Despite these properties, current evidence regarding the behavior and long-term clinical stability of graphene-modified PMMA materials under simulated intraoral aging conditions remains limited.

Thermal aging represents one of the most commonly used in vitro approaches to simulate intraoral environmental fluctuations. Repeated temperature changes may induce hydrothermal degradation in polymer-based materials through mechanisms such as water diffusion, matrix plasticization, and interfacial degradation within composite systems. These processes can affect the optical properties, surface integrity, and mechanical stability of restorative materials over time. Therefore, evaluating the aging behavior of contemporary CAD/CAM polymer materials is essential for predicting their long-term clinical performance.

Because polymer-based CAD/CAM materials differ in structural composition and reinforcement strategies, their comparative evaluation is important for understanding their long-term clinical behavior. In particular, polyetheretherketone (PEEK), fiber-reinforced composite polymers, and graphene-reinforced PMMA represent distinct material design approaches within polymer-based restorative systems. Therefore, evaluating their physicochemical and biological behavior under standardized aging conditions may provide valuable insights into their relative performance and potential applications in prosthetic dentistry.

Previous studies have primarily focused on mechanical parameters such as flexural strength and fracture resistance of high-performance CAD/CAM polymers [3,4,7,10]. However, comprehensive studies that simultaneously evaluate optical performance, surface properties, mechanical behavior, structural integrity, and biological compatibility under a standardized thermal aging protocol remain limited for PEEK, fiber-reinforced composite polymers, and graphene-reinforced PMMA materials. Consequently, current evidence regarding the physicochemical behavior and biological safety of these contemporary polymer-based materials in long-term prosthetic restorations remains limited. The development of polymer-based CAD/CAM materials has expanded metal-free treatment options in digital dentistry. However, comparative evidence evaluating new-generation polymer-based materials under standardized simulated intraoral aging conditions remains insufficient. Thus, this study aimed to compare the effects of thermal aging on three CAD/CAM polymer-based dental materials—polyether ether ketone (PEEK), fiber-reinforced composite polymer (FRC), and graphene-reinforced PMMA polymer (G-CAM) with respect to color stability, surface roughness, microhardness, structural properties, and cytotoxicity. The null hypothesis was that thermal aging would not result in statistically significant differences in color stability, surface roughness, microhardness, structural characteristics, or cytotoxicity among the tested CAD/CAM polymer-based dental materials.

## 2. Materials and Methods

### 2.1. Study Design

This in vitro experimental study was conducted to evaluate the effects of thermal aging on three CAD/CAM polymer-based dental materials: polyetheretherketone (PEEK; Juvora Ltd., Thornton-Cleveleys, UK), a fiber-reinforced composite polymer (Trilor; Bioloren, Saronno, Italy), and a graphene-reinforced PMMA-based polymer (G-CAM; Graphenano Dental, Valencia, Spain). Detailed information regarding the tested materials is presented in Table 1. An a priori sample size calculation was performed using G*Power software (version 3.1; Heinrich-Heine-University Düsseldorf, Düsseldorf, Germany). Based on a significance level of α = 0.05, a statistical power of 80%, and a moderate effect size (f = 0.35), the required sample size was calculated to be 9 specimens per material group. The calculation was primarily based on Vickers microhardness values, which served as the primary mechanical parameter evaluated in this study. A total of 27 disk-shaped specimens were fabricated (*n* = 9 per material) under standardized conditions. Before aging procedures, all specimens were stored in distilled water at 37 °C for 24 h to allow initial water equilibration. Color stability, surface roughness, microhardness, structural characteristics, and cytotoxicity were evaluated before and after thermal aging.

### 2.2. Specimen Preparation

**Figure 1:**
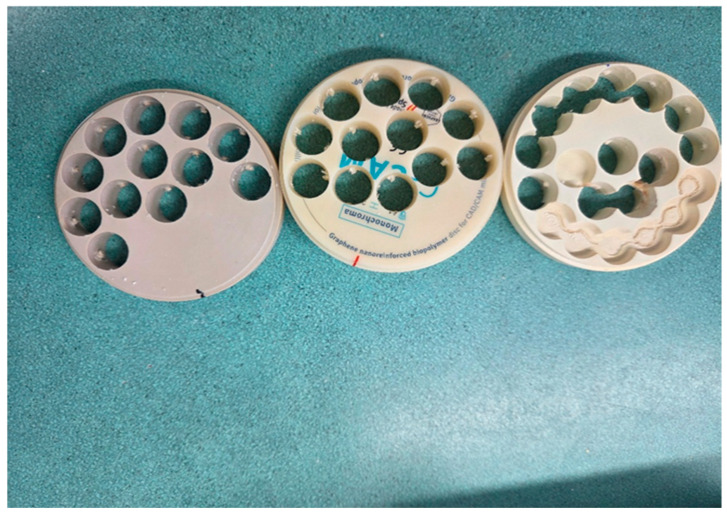
Polymer-based CAD/CAM blocks used for specimen fabrication.

Disk-shaped specimens were digitally designed using EXOCAD DentalCAD software (version 3.1; exocad GmbH, Darmstadt, Germany) with a diameter of 10 mm and a thickness of 2 mm. The design files were exported as STL files and transferred to the milling unit using WorkNC Dental CAM software (version 2021.1.2105; Hexagon Manufacturing Intelligence, UK). The specimens were milled from pre-manufactured CAD/CAM blocks using a computer-controlled milling unit (Redon Hybrid, Redon Technology, Istanbul, Türkiye) according to the manufacturer’s instructions. The final specimen dimensions were verified using a digital caliper (Hi-Wendy, New Taipei City, Taiwan) to ensure dimensional standardization. All specimens were sequentially ground using 600- and 800-grit silicon carbide (SiC) abrasive papers under water irrigation, followed by polishing with a standardized polishing paste (Universal Polishing Paste, Ivoclar Vivadent, Schaan, Liechtenstein) applied under consistent manual pressure to obtain comparable surface conditions before testing. The CAD/CAM blocks used for specimen fabrication are shown in Figure 1.

### 2.3. Thermal Cycling

**Figure 2:**
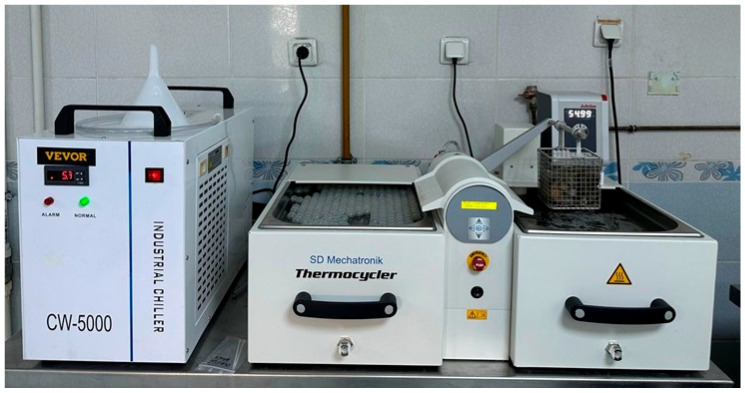
Automatic thermocycler used for thermal aging procedure.

Thermal cycling was performed using an automatic thermocycler (Thermocycler 1100/1200, SD Mechatronik, Feldkirchen-Westerham, Germany) (Figure 2). All specimens were subjected to 10,000 thermal cycles between 5 °C and 55 °C in distilled water to simulate approximately one year of intraoral clinical aging [13].

Each thermocycling cycle consisted of a 30 s dwell time in each water bath and a 10 s transfer time between baths, following commonly used thermocycling protocols in dental materials research to simulate intraoral thermal fluctuations and ensure methodological comparability among studies [13,14]. To maintain stable experimental conditions, the water reservoirs were periodically refilled to compensate for evaporation, and the thermocycling system was continuously monitored to ensure consistent temperature regulation and uninterrupted specimen transfer throughout the aging process.

### 2.4. Color Stability Measurements

Color measurements were performed before and after thermal aging using a spectrophotometer (VITA Easyshade V, VITA Zahnfabrik, Bad Säckingen, Germany). All measurements were conducted in the same room under standardized lighting conditions, using a color evaluation booth equipped with D65 daylight illumination at 1500 lux. The spectrophotometer was calibrated before each measurement using the calibration block integrated into the charging unit according to the manufacturer’s instructions. Specimens were positioned against a neutral white background, and the measuring probe was placed perpendicular to the specimen surface at the central region. For each specimen, three consecutive measurements were recorded before thermal aging, and three additional measurements were obtained after thermal aging from the same central location. Mean L*, a*, and b* values were calculated for both conditions, and color differences (ΔE_00_) were determined using the CIEDE2000 color-difference formula [15,16].

### 2.5. Surface Roughness Measurements

Surface roughness (Ra) measurements were performed before and after thermal aging using a non-contact 3D optical profilometer (Profilm 3D, Filmetrics, San Diego, CA, USA). A 20× objective epi-fluorescence lens was used, and scans were obtained over an area of 1.9 × 1.6 mm^2^ located at the center of each specimen. The profilometer works on the principle of White Light Interferometry (WLI) (FILMETRICS, A KLA Company, San Diego, CA, USA). In this technique, broadband white light illuminates both the test surface and a reference mirror. The reflected beams from the reference and specimen generate constructive and destructive interference patterns that are detected by a charge-coupled device (CCD) image sensor and digitally processed to reconstruct the surface topography. The Profilm 3D system can calculate 47 standardized ASME/EUR/ISO surface roughness parameters and measure surface features within a vertical range of 50 nm to 10 mm.

In the present study, surface roughness was quantified using the Ra parameter (arithmetical mean surface roughness) derived from three-dimensional surface topography analysis. For each specimen, measurements were obtained from three different surface regions, and the mean Ra value (µm) was calculated as the average of the three readings. All measurements were performed under controlled laboratory conditions by the same operator to minimize measurement variability.

### 2.6. Surface Microhardness Measurement

Surface microhardness was evaluated before and after thermal aging using a digital microhardness tester (HVS-1000, Qingdao, China). A load of 1000 gf (9.8 N) was applied to each specimen surface for 30 s. The Vickers hardness number (VHN) was recorded for analysis. The measurements were performed in accordance with the ASTM E384 standard for microindentation hardness testing [17,18]. All measurements were performed under standardized laboratory conditions by the same operator.

### 2.7. Spectroscopic Analyses (FTIR and Raman)

Spectroscopic analyses were performed before and after thermal aging to evaluate potential chemical and structural alterations in the tested materials. Raman spectroscopy was conducted using a Raman spectrophotometer (NRS-4500, Jasco, Tokyo, Japan), and Fourier-transform infrared (FTIR) analysis was performed using an FTIR spectrometer (FT/IR-6X, Jasco, Tokyo, Japan). All measurements were carried out according to the manufacturer’s recommended operating conditions.

### 2.8. Surface Morphology (SEM) Analysis

Surface morphology of the specimens was evaluated before and after thermal aging using a field-emission scanning electron microscope (FE-SEM; Hitachi SU5000, Tokyo, Japan). Before examination, the specimens were sputter-coated with a thin gold layer to improve surface conductivity.

### 2.9. Cytotoxicity Analysis

Sample extracts were prepared in accordance with ISO 10993-12 (Biological evaluation of medical devices—Sample preparation and reference materials) [19]. Briefly, specimens were disinfected in 70% ethanol for 5 min and rinsed with sterile deionized water. The samples were then immersed in Dulbecco’s Modified Eagle Medium (DMEM) supplemented with 10% fetal bovine serum (FBS) and 1% penicillin–streptomycin (PS). The extraction ratio was adjusted to 6 cm^2^/mL based on the measured surface area of each specimen. Natural rubber latex extracts prepared under identical conditions served as the positive control. All samples were incubated at 37 °C for 72 h, and the obtained extracts were used immediately after preparation.

Human gingival fibroblast (HGF) cells (PCS-201-018™, ATCC, Manassas, VA, USA) were used for cytotoxicity evaluation. Cells (passage 7) were thawed at 37 °C, counted using an automated cell counter (TC20, Bio-Rad, Hercules, CA, USA), and cultured in DMEM supplemented with 10% FBS and 1% PS under standard conditions (37 °C, 5% CO_2_). Real-time cell analysis (RTCA) was performed using the xCELLigence RTCA DP system with 16-well E-plates (Agilent Technologies, Inc., Santa Clara, CA, USA). Initially, 100 µL of culture medium was added to each well to obtain background impedance readings. Cells were then seeded at a density of 1 × 10^4^ cells/well to achieve a final volume of 200 µL. After allowing cell attachment for 24 h, the culture medium was replaced with undiluted sample extracts added in triplicate wells. Cell viability was continuously measured for an additional 72 h.

### 2.10. Statistical Analysis

Surface roughness, color change, and surface microhardness values were analyzed using one-way analysis of variance (ANOVA) to determine significant differences among the study groups. Tukey’s HSD post hoc comparisons were performed to identify pairwise differences. Pre-aging and post-aging comparisons within the groups were performed using *t*-tests. Statistical significance was set at *p* < 0.05. Data analysis was conducted using SPSS (version 25.0). Cytolysis rates were calculated using xCELLigence RTCA software (version 2.0).

## 3. Results

### 3.1. Color Stability

**Figure 3:**
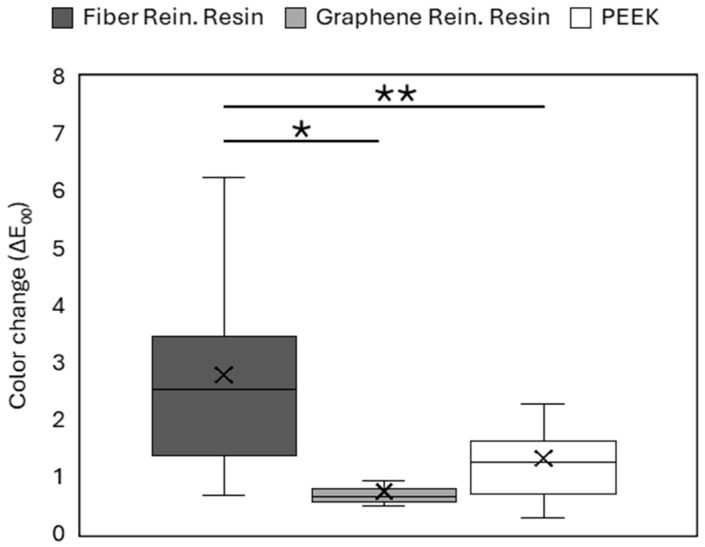
Color change (ΔE_00_) values of the materials before and after thermal aging. (* and ** denote statistically significant difference).

The color stability of the polymer-based CAD/CAM materials was evaluated before and after thermal aging. The color change values (ΔE_00_) obtained for each material are presented in Figure 3.

All tested materials exhibited measurable color changes following thermal aging. The magnitude of color change differed among the materials, indicating material-dependent responses to the aging procedure. The highest color change values were observed in the FRC group, whereas the lowest values were recorded in the G-CAM group. The G-CAM group demonstrated significantly lower color change values than the other materials (*p* < 0.05), whereas no statistically significant difference was observed between the FRC and PEEK groups (*p* > 0.05).

### 3.2. Surface Roughness

**Figure 4:**
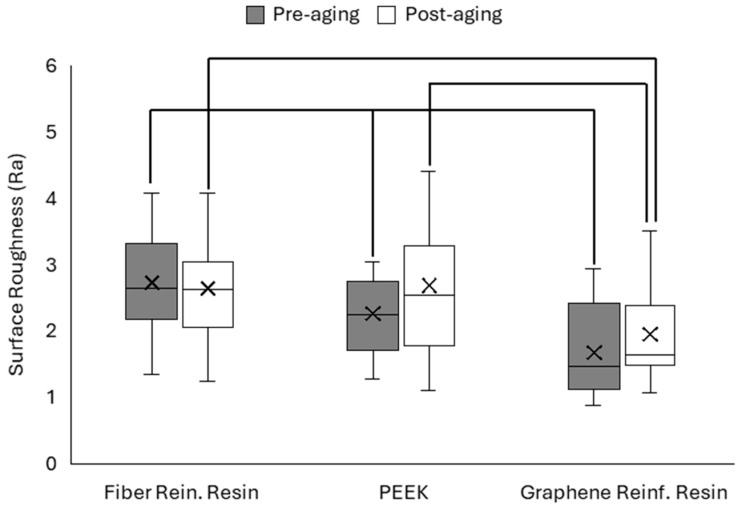
Mean surface roughness (Ra) values of the tested materials before and after thermal aging.

Surface roughness (Ra) of the polymer-based CAD/CAM materials was measured using an optical profilometer before and after thermal aging. The mean Ra values for each material are presented in Figure 4.

One-way ANOVA revealed statistically significant differences among the materials before aging (*p* < 0.05). The FRC group exhibited the highest roughness values, whereas the G-CAM group showed the lowest values. After thermal aging, no statistically significant difference was observed between the FRC and PEEK groups (*p* > 0.05), whereas the G-CAM group maintained significantly lower roughness values than the other materials (*p* < 0.05). Paired *t*-test analysis demonstrated no statistically significant differences between pre-aging and post-aging measurements within any material group (*p* > 0.05).

### 3.3. Surface Microhardness

**Figure 5:**
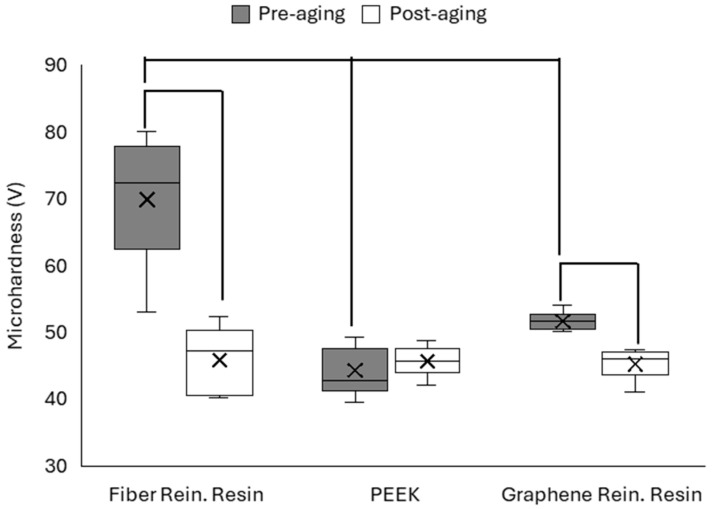
Mean Vickers microhardness (VHN) values of the tested materials before and after thermal aging.

Vickers microhardness values of the polymer-based CAD/CAM materials were measured before and after thermal aging. The mean hardness values obtained for each material are presented in Figure 5.

One-way ANOVA revealed statistically significant differences among the materials before aging (*p* < 0.05), with the FRC group exhibiting the highest and the G-CAM group exhibiting the lowest surface microhardness values. After thermal aging, statistically significant differences among the material groups were maintained (*p* < 0.05). The G-CAM group exhibited lower microhardness values than the other materials. Paired *t*-test analysis indicated no statistically significant differences in the PEEK group between pre-aging and post-aging measurements (*p* > 0.05). However, a statistically significant decrease in microhardness was observed in both the FRC and G-CAM groups after thermal aging (*p* < 0.05).

### 3.4. Spectroscopic Analysis (FTIR and Raman)

**Figure 6:**
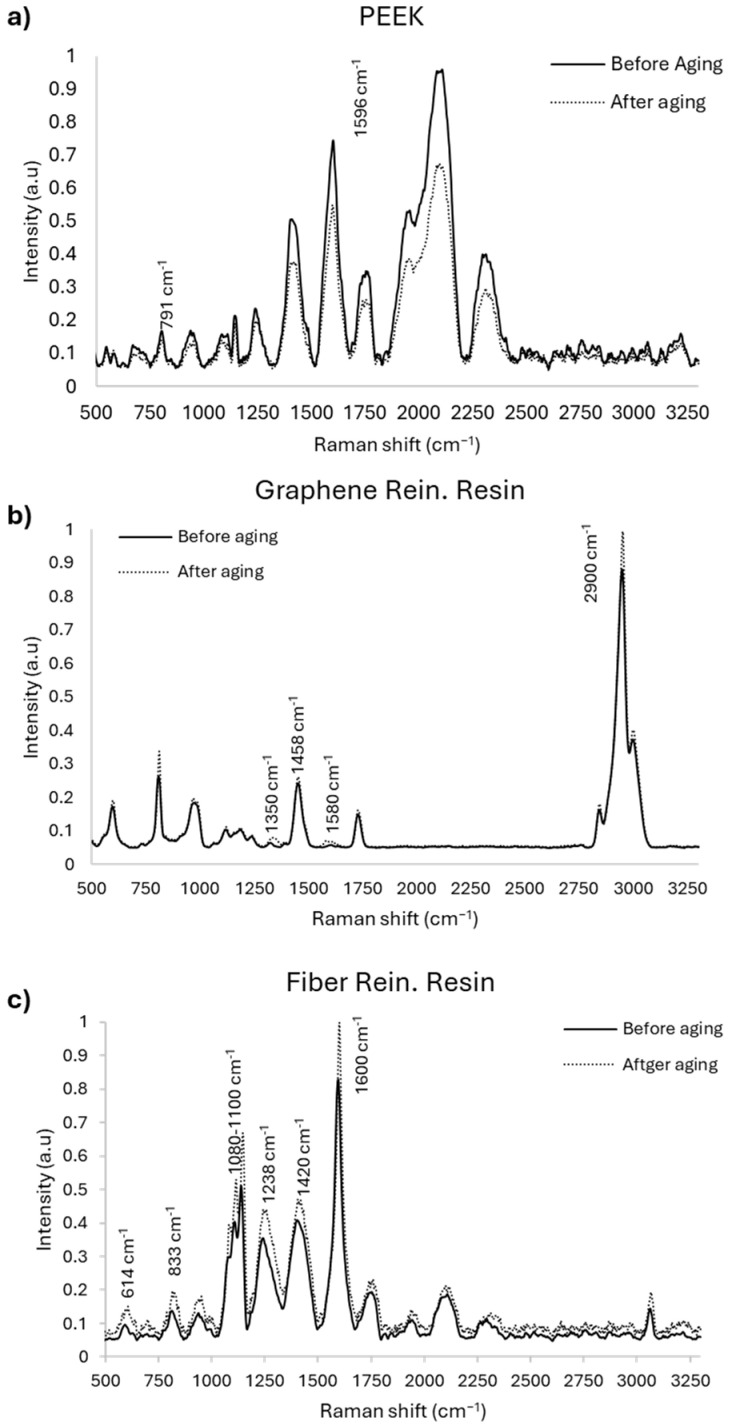
Representative FTIR and Raman spectra of the materials before and after thermal aging.

The chemical and molecular characteristics of the polymer-based CAD/CAM materials were evaluated using Fourier-transform infrared spectroscopy (FTIR) and Raman spectroscopy before and after thermal aging. Representative spectra obtained for each material are presented in Figure 6.

FTIR analysis revealed that the characteristic absorption bands of the tested materials were preserved after thermal aging. No marked shifts or disappearance of major functional group peaks were observed when pre- and post-aging spectra were compared. Similarly, Raman spectroscopic analysis demonstrated characteristic Raman bands for each material both before and after thermal aging. Comparison of the spectra showed no substantial changes in peak positions or overall spectral profiles following aging.

### 3.5. Surface Morphology (SEM)

**Figure 7:**
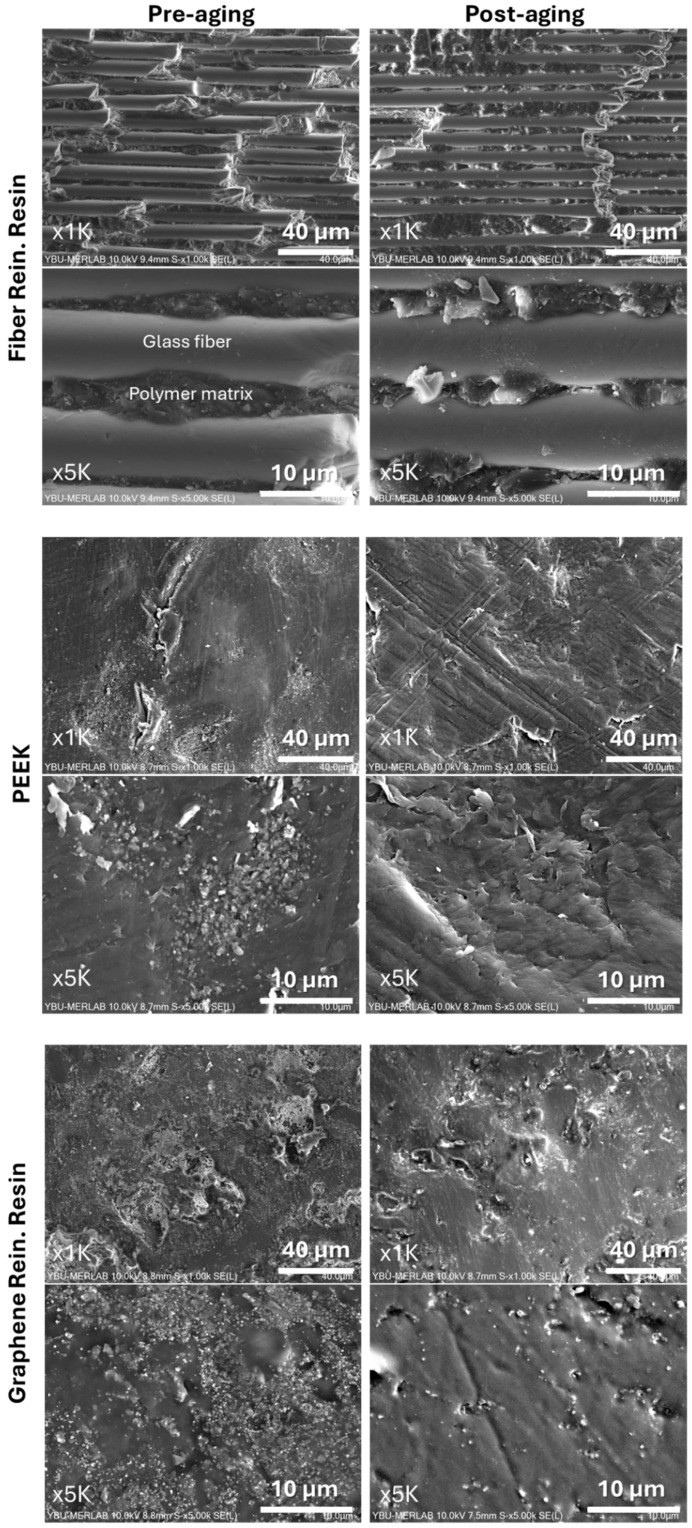
SEM micrographs illustrating the surface morphology of the tested materials before and after thermal aging at two magnifications (×1000 and ×5000).

The surface morphology of the polymer-based CAD/CAM materials was evaluated using scanning electron microscopy (SEM) before and after thermal aging. Representative SEM micrographs obtained from each material group are presented in Figure 7.

Before thermal aging, the surfaces of the tested materials exhibited relatively homogeneous morphologies with material-specific surface features. Following thermal aging, changes in surface texture were observed, including variations in surface uniformity and the presence of surface irregularities in certain materials. Comparison of the pre- and post-aging SEM images demonstrated material-dependent differences in surface morphology.

### 3.6. Cytotoxicity

**Figure 8:**
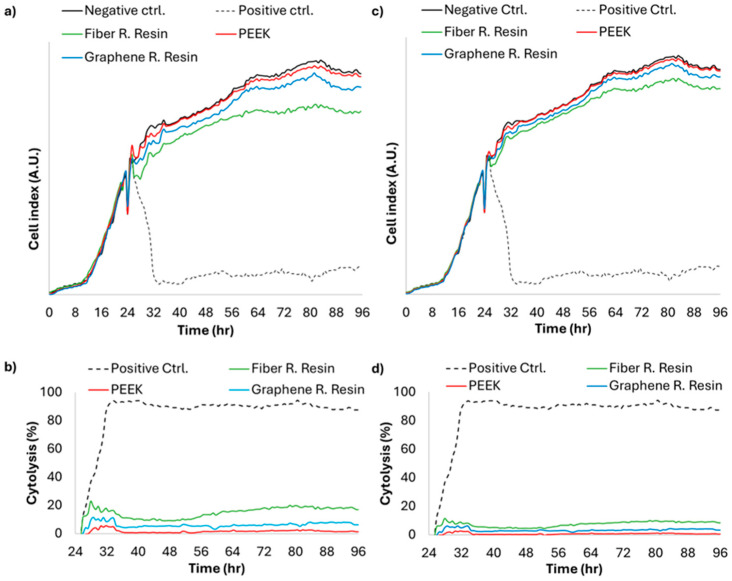
Pre-aging (**a**,**b**) and post-aging (**c**,**d**) time-dependent cell index (**a**,**c**) and percent cytolysis (**b**,**d**) obtained from xCELLigence RTCA.

The cytotoxicity of the tested materials was evaluated using the xCELLigence real-time cell analysis (RTCA) system before and after thermal aging. The cell viability results obtained for each material are presented in Figure 8.

All tested materials exhibited acceptable cytocompatibility profiles, with cell viability values well above the 70% threshold established in the ISO 10993 standard. Extracts obtained from PEEK and graphene-reinforced resin samples yielded viability values comparable to those of the negative control group both before and after aging. A slight decrease in viability, to approximately 80%, was observed in extracts from fiber-reinforced resin samples before aging. Although this decrease remained above the 70% threshold, the difference was observable. After aging, the reduction in viability was less pronounced than that observed before aging, with approximately 90% viability at the lowest level. A steep decrease in cell viability to approximately 10% was observed in the positive control samples, indicating the internal consistency of the analysis.

## 4. Discussion

Rapid advances in digital dentistry have increased the use of CAD/CAM technologies in the production of prosthetic restorations. CAD/CAM systems have not only improved production precision but also enabled the clinical use of high-performance polymer-based materials as metal-free aesthetic alternatives to conventional prosthetic base materials. These materials have attracted attention because of their elastic behavior, lower brittleness compared with ceramics, biomechanical compatibility with oral tissues, and shock-absorbing properties that better mimic natural tooth structure [7,20,21]. Unlike conventionally produced materials, CAD/CAM materials are industrially manufactured from pre-polymerised blocks under standard production conditions. Thus, it has been reported that they exhibit greater structural consistency than materials traditionally processed in the laboratory [22]. Furthermore, previous studies have shown that exposure to water and thermal cycling can affect the surface properties and mechanical stability of PEEK materials and have emphasised the importance of aging resistance for long-term clinical performance [9]. Consequently, understanding how different reinforcement strategies affect aging behavior has become a key focus in prosthetic material research.

In this study, the effects of thermal aging on the optical, surface, mechanical, structural, and biological behaviour of three CAD/CAM polymer-based dental materials were evaluated. Thermal aging influenced material properties differently among the tested materials. Changes mainly involved colour stability, surface properties, and microhardness before and after aging, whereas the chemical structure remained stable. Therefore, the null hypothesis that thermal aging would not affect the evaluated properties was partially rejected.

Polymer restorative materials exhibit physicochemical changes after exposure to temperature fluctuations and moisture conditions that simulate the oral environment. Water sorption by polymer materials may cause plasticization, which can alter intermolecular spacing and thereby affect physical properties over time [9]. Moreover, CAD/CAM materials fabricated from pre-polymerized polymer blocks exhibit greater structural homogeneity than conventionally processed polymer materials [22].

In the present study, the different responses observed among the tested materials indicate that aging behavior is largely influenced by structural design rather than manufacturing technique alone. Semicrystalline polymers such as PEEK can limit excessive water penetration because crystalline regions act as stabilizing areas. In contrast, composite systems depend on filler–matrix interactions and interfacial integrity to preserve performance during aging procedures [20,23]. Similarly, graphene-reinforced polymers exhibit behavior influenced by nanoscale reinforcement efficiency and dispersion quality rather than bulk polymer composition alone [24].

Color stability is an important factor for the long-term clinical success of polymer-based CAD/CAM restorative materials, particularly in esthetic restorations. During thermal aging, polymer materials may show color changes related to water sorption and internal structural relaxation. Pigment diffusion within the material can further influence optical appearance over time. The degree of discoloration depends on material composition, crystallinity, and reinforcement strategy. PEEK materials generally maintain stable color behavior, which has been linked to their semi-crystalline structure and low water sorption characteristics that help limit hydrolytic degradation and pigment penetration [9,25]. The findings of the present study are in agreement with these observations. Consistent with these findings, material-dependent differences in color change were observed following thermocycling. Among the evaluated materials, the graphene-reinforced polymer (G-CAM) demonstrated the lowest color change values. This behavior may be associated with the barrier effect of graphene nanosheets, which can restrict water diffusion and reduce chromophore penetration within the polymer matrix. Similar improvements in resistance to environmental degradation and staining have been reported for resin-based systems exposed to thermal and staining challenges [26]. In contrast, fiber-reinforced composite materials showed greater color change. This finding may be related to interfacial regions between reinforcing fibers and the polymer matrix. These areas can facilitate water uptake and promote microstructural heterogeneity during aging. Previous studies have indicated that optical aging behavior in reinforced polymers is influenced mainly by reinforcement architecture and matrix stability rather than material classification alone [11,27].

Most importantly, surface roughness does not always correlate directly with color stability. Previous studies on PEEK-based materials have indicated that surface finishing methods may affect surface topography and optical properties; however, the relationship between surface roughness and color change appears to be material-specific [28]. In the present study, color change was mainly associated with intrinsic material properties rather than surface texture alone. According to the perceptibility and acceptability thresholds proposed by Paravina et al., the color changes observed after thermal aging remained within clinically acceptable limits [29]. Statistically significant differences were detected among the materials. The present findings indicate that polymer architecture and reinforcement strategy play important roles in maintaining the optical stability of contemporary CAD/CAM polymer restorative materials.

Surface roughness plays an important role in plaque accumulation, staining susceptibility, and long-term biological compatibility. Thermal cycling may induce repeated expansion–contraction stresses, leading to microstructural changes at the material surface. Previous studies evaluating CAD/CAM polymers have shown that industrial polymerization processes reduce internal defects and surface irregularities, improving resistance to aging-related roughness changes [9,22].

Surface alterations remained limited after thermal aging in the present study. PEEK materials maintained relatively smooth surfaces, which can be attributed to their semicrystalline structure, chemical inertness, and low water sorption, which help reduce hydrothermal degradation [9,21]. In contrast, fiber-reinforced composite materials showed greater surface variability, likely related to fiber exposure and matrix–fiber interfacial degradation during aging, as previously described for reinforced polymer systems [11,27].

Graphene-reinforced polymers may also benefit from nanoscale reinforcement mechanisms. Graphene nanosheets can modify surface energy, limit crack propagation, and act as diffusion barriers to water penetration, thereby improving surface stability under thermal stress [13]. SEM observations in the present study supported these mechanisms, showing material-dependent morphological features after aging without evidence of pronounced surface deterioration.

Comparison of the pre-aging and post-aging SEM images revealed no major structural alterations in the material surfaces. The observed surface features remained generally similar before and after thermal aging. This observation is consistent with the surface roughness measurements, which showed no statistically significant differences between pre-aging and post-aging values. Therefore, the SEM findings support the profilometric results, indicating that thermal aging did not produce substantial changes in surface morphology.

From a clinical perspective, maintaining low surface roughness is important for minimizing biofilm retention and supporting restoration longevity. Previous studies have shown that smoother restorative surfaces are associated with reduced bacterial adhesion and improved long-term clinical performance of polymer-based restorations [30]. In the present study, the limited changes in roughness observed after aging indicate the favorable resistance of contemporary CAD/CAM polymer materials.

Surface microhardness is an important mechanical parameter reflecting the resistance of restorative materials to localized deformation, wear, and long-term functional degradation under intraoral conditions. In the present study, material-dependent behavior was observed after thermal aging. Before aging, fiber-reinforced composite (FRC) materials showed the highest microhardness values, whereas PEEK exhibited comparatively lower hardness. The higher initial hardness of FRC materials can be attributed to the reinforcing effect of embedded fibers, which improve stress distribution and indentation resistance by enabling effective load transfer within the composite structure. Similar mechanical behavior has been reported for fiber-reinforced CAD/CAM polymers, in which reinforcement architecture influences initial hardness [7,11,31].

Despite its lower baseline hardness, PEEK showed no statistically significant change after thermal aging, demonstrating resistance to hydrothermal degradation. This behavior can be explained by the semicrystalline structure and chemical stability of PEEK, together with its low water sorption characteristics, which limit polymer chain mobility under cyclic thermal stress. In the present study, these features likely contributed to the preservation of mechanical properties after aging. Similar mechanical stability of PEEK compared with multiphase composite systems has also been reported in previous dental research [8,9,32,33].

In contrast, both fiber-reinforced and graphene-reinforced polymer materials showed significant reductions in microhardness following thermocycling. Thermal aging may promote water sorption, matrix plasticization, and degradation at the reinforcement–matrix interfaces, thereby facilitating microcrack formation and weakening stress-transfer mechanisms. Reinforced polymer systems are particularly susceptible to hydrothermal fatigue because interfacial regions between fillers or fibers and the polymer matrix act as preferential pathways for moisture diffusion and structural relaxation. Comparable aging-related decreases in hardness have been reported for CAD/CAM composite materials exposed to thermocycling and simulated oral conditions [7,34,35].

Graphene reinforcement introduces nanoscale strengthening mechanisms that may enhance initial mechanical performance by improving stress transfer and modifying surface energy. However, aging processes can reduce reinforcement efficiency due to moisture ingress, interlayer separation of graphene platelets, and interfacial degradation within the polymer network. Experimental studies on graphene-modified dental polymers have shown that although graphene incorporation may initially improve mechanical properties, thermocycling can reduce Vickers microhardness due to structural rearrangement and hydrothermal instability [10,36,37].

Comparing post-aging values, no statistically significant differences were found among the groups, suggesting that thermal aging may contribute to partial homogenization of surface mechanical properties despite initial differences in hardness. These findings suggest that the reinforcement strategy primarily influences baseline mechanical strength, whereas long-term performance depends largely on the intrinsic stability of the polymer matrix. The present results are consistent with previous studies reporting that thermoplastic materials such as PEEK tend to better maintain hardness values, whereas composite-based materials appear more susceptible to hydrothermal aging due to fiber–matrix interfacial interactions and moisture diffusion pathways [11]. From a clinical perspective, maintaining hardness stability may contribute to long-term wear resistance of CAD/CAM polymer materials exposed to repeated thermal fluctuations.

The higher variability observed in the microhardness values of the fiber-reinforced composite compared with PEEK and the graphene-reinforced resin can be attributed to the heterogeneous microstructure of fiber-reinforced polymer systems at the micro-scale. During microhardness testing, the indentation area may interact either predominantly with the polymer matrix or partially with reinforcing fibers or fiber–matrix interfacial regions. As a result, the local resistance to indentation can vary depending on the microstructural region probed by the indenter. In contrast, PEEK is a relatively homogeneous semicrystalline thermoplastic polymer with a more uniform microstructure. Similarly, graphene-reinforced PMMA systems contain nanoscale fillers that are more uniformly dispersed within the polymer matrix, producing a comparatively homogeneous mechanical response at the microscale.

FTIR and Raman analyses revealed no significant shifts, disappearances, or alterations in characteristic peaks, indicating that the tested materials retained chemical and molecular stability after thermal aging. In the present study, cytotoxicity findings were consistent with this stability, as preserved molecular structures were associated with sustained cell viability. The stability observed in PEEK can be attributed to its semi-crystalline polyaryletherketone backbone, which provides resistance to hydrolysis and oxidation. Previous studies have also reported spectral stability of PEEK after autoclaving or thermal cycling, with no detectable phase changes in FTIR analysis [38,39]. Limited surface oxidation under severe acid–thermal conditions has been shown to slightly increase crystallinity without affecting bulk spectral characteristics [39]. Clinically, these findings suggest reliable long-term performance of PEEK when used as a CAD/CAM abutment material.

Studies investigating graphene- and glass fiber-modified dental resins have similarly reported spectroscopic stability after thermal exposure, with preserved FTIR and Raman signatures and no significant changes in the degree of conversion [40,41,42]. However, higher graphene concentrations have been associated with minor spectral shifts related to oxidative stress rather than bulk chemical degradation [41]. These observations support the molecular stability of graphene- and fiber-reinforced polymer systems following thermal aging. The present findings suggest that thermal aging primarily affected physical and interfacial properties without inducing detectable chemical changes in the polymer matrices.

The cytotoxicity findings demonstrated that all evaluated CAD/CAM polymer materials exhibited acceptable cytocompatibility both before and after thermal aging. Cell viability values remained above the 70% threshold defined by ISO 10993-5 [43]. confirming the biological safety of the tested materials under simulated oral aging conditions.

The high cytocompatibility observed for PEEK can be attributed to its chemically inert polyaryletherketone structure, low solubility, and minimal release of degradation products. These characteristics limit interactions between residual substances and cellular metabolism, supporting stable biological performance. Previous investigations have similarly reported negligible cytotoxic, mutagenic, and immunogenic responses associated with PEEK materials [44,45,46].

The fiber-reinforced CAD/CAM polymer exhibited slightly lower cell viability prior to thermal aging, which may be attributable to initial leachable components from the fiber–matrix interface. Interfacial regions in fiber-reinforced systems may temporarily influence early cellular responses through the limited release of residual substances. However, the improvement in viability following thermocycling suggests material stabilization and reduction in surface reactivity after aging, consistent with findings reported for industrially polymerized composite systems [47,48,49].

The graphene-reinforced polymer exhibited cytocompatibility comparable to the negative control both before and after aging, indicating that graphene incorporation at appropriate concentrations does not compromise biological safety. Graphene fillers have been reported to enhance mechanical performance while maintaining favorable cellular responses when homogeneously dispersed within polymer matrices. The stable cell viability observed after aging suggests that graphene modification did not promote cytotoxic degradation under simulated oral conditions [50].

The comparable cytocompatibility levels observed among all materials after aging align with the FTIR and Raman spectroscopic findings demonstrating preserved molecular structures. Since biological response is closely related to chemical stability, the absence of detectable molecular degradation supports sustained cellular compatibility of the tested polymers.

The present study evaluated optical, surface, mechanical, and biological properties and potential relationships among these properties should also be considered. Surface characteristics may influence optical behavior because increased surface roughness can enhance light scattering and staining susceptibility. However, in the present study, the limited changes observed in surface roughness suggest that the color changes were more strongly associated with intrinsic material composition rather than surface topography alone. The Ra values measured before and after thermal aging did not show statistically significant alterations within the material groups, indicating that surface texture remained relatively stable during the aging process. Therefore, the color differences observed after thermocycling are more likely related to intrinsic material characteristics such as polymer matrix composition, filler or reinforcement structure, and water sorption behavior, which may influence internal light transmission. Similarly, hydrothermal aging mechanisms such as water sorption and matrix plasticization may simultaneously affect surface integrity and mechanical behavior, explaining the decrease in microhardness observed in reinforced polymer systems. The considerable reduction in the glass fiber-reinforced system may be attributed to hydrothermal degradation at the fiber-matrix interface. The more moderate change in the graphene-reinforced system may be related to the nanoscale reinforcement provided by graphene platelets, which can better improve load distribution within the polymer. The stable microhardness observed in PEEK can be attributed to the semicrystalline structure and inherent chemical resistance of PEEK. Furthermore, the preserved molecular structures detected by FTIR and Raman spectroscopy are consistent with the stable cytocompatibility results, indicating that the absence of chemical degradation may contribute to maintaining favorable biological responses.

The findings of this study suggest that high-performance CAD/CAM polymer materials, including PEEK, fiber-reinforced composites, and graphene-reinforced polymers, can maintain acceptable biological performance under thermal cycling. However, additional studies involving longer aging periods and broader biological evaluations are required to better understand possible long-term degradation behavior under clinical conditions.

Overall, the present study demonstrated that thermal aging produced material-dependent effects on the physicochemical and biological properties of CAD/CAM polymer-based materials while preserving their structural integrity and biocompatibility. Surface roughness, color stability, microhardness, spectroscopic analyses, and cytotoxicity findings indicated stable material behavior under simulated oral aging conditions. The absence of significant molecular alterations observed in FTIR and Raman analyses supports the chemical stability of these materials following aging.

Changes in color stability, surface roughness, and microhardness should be interpreted together when evaluating the aging behavior of polymer-based restorative materials. Previous studies have reported that water absorption, pigment degradation, and surface degradation may contribute to discoloration in denture base polymers, while increased surface roughness may facilitate stain accumulation and influence the optical appearance of restorative materials over time [9,51]. In addition, reductions in surface hardness may reflect structural relaxation or degradation within the polymer matrix following hydrothermal aging [9]. Therefore, the combined evaluation of optical, surface, and mechanical parameters provides a more comprehensive understanding of the long-term performance of CAD/CAM polymer-based materials under simulated intraoral conditions and may provide useful insights for selecting appropriate materials for long-term prosthetic applications.

A limitation of the present study is that only thermal aging was evaluated under controlled laboratory conditions. Therefore, the findings may not fully reflect the complex intraoral environment, where mechanical loading, pH fluctuations, and microbial activity may also influence the long-term behavior of restorative materials.

## 5. Conclusions

Within the limitations of this in vitro study, the effects of thermal aging on CAD/CAM polymer-based dental materials were found to be material-dependent.

PEEK demonstrated the highest microhardness stability and maintained favorable surface characteristics after thermal aging, indicating relatively higher resistance to hydrothermal degradation.

Fiber-reinforced composite (Trilor) exhibited moderate changes in surface and mechanical properties after aging, which may be related to the behavior of the polymer matrix and fiber–matrix interactions under thermal stress.

Graphene-reinforced polymer (G-CAM) showed acceptable color stability and cytocompatibility, although greater variations in some surface and mechanical parameters were observed after aging.

Overall, all tested materials preserved acceptable physicochemical and biological performance under simulated oral aging conditions.

Further long-term aging simulations and clinical studies are required to confirm the long-term clinical performance of these CAD/CAM polymer-based materials.

## Figures and Tables

**Table 1:** CAD/CAM polymer-based materials used in the study.

Material Type	Commercial Name	Manufacturer
Fiber-reinforced composite (FRC) polymer	Trilor	Bioloren S.r.l., Saronno, Italy
Polyether ether ketone (PEEK)	JUVORA	JUVORA Ltd., Thornton-Cleveleys, UK
Graphene-reinforced PMMA-based acrylic resin G-CAM	G-CAM	Graphenano Dental, Valencia, Spain

## Data Availability

The original contributions presented in this study are included in the article. Further inquiries can be directed to the corresponding author.
